# Neural Network Repair of Lossy Compression Artifacts in the September 2015 to March 2016 Duration of the MMS/FPI Data Set

**DOI:** 10.1029/2019JA027181

**Published:** 2020-04-24

**Authors:** Daniel da Silva, A. Barrie, D. Gershman, S. Elkington, J. Dorelli, B. Giles, W. Patterson

**Affiliations:** ^1^ Whiting School of Engineering Johns Hopkins University Baltimore MD USA; ^2^ NASA Goddard Spaceflight Center Greenbelt MD USA; ^3^ Trident Vantage Systems Greenbelt MD USA; ^4^ Laboratory for Atmospheric and Space Physics University of Colorado Boulder Boulder CO USA; ^5^ Aurora Engineering Potomac MD USA

**Keywords:** Magnetosphere, Neural Networks, Image Processing, Magnetic Reconnection

## Abstract

During the September 2015 to March 2016 duration (sometimes referred to as Phase 1A) of the Magnetospheric Multiscale Mission, the Dual Electron Spectrometers (DES) were configured to generously utilize lossy compression. While this maximized the number of velocity distribution functions downlinked, it came at the expense of lost information content for a fraction of the frames. Following this period of lossy compression, the DES was reconfigured in a way that allowed for 95% of the frames to arrive to the ground without loss. Using this high‐quality set of frames from on‐orbit observations, we compressed and decompressed the frames on the ground to create a side‐by‐side record of the compression effect. This record was used to drive an optimization method that (a) derived basis functions capable of approximating the lossless sample space and with nonnegative coefficients and (b) fitted a function which maps the lossy frames to basis weights that recreate the frame without compression artifacts. This method is introduced and evaluated in this paper. Data users should expect a higher level of confidence in the absolute scale of density/temperature measurements and notice less sinusoidal bias in the velocity X and Y components (GSE)

## Key Points

- 57% of the burst resolution frames in the September 2015 to March 2016 duration of the MMS mission were affected by some form of lossy compression error
- This data can be corrected by optimizing a model with side‐by‐side examples of the before/after compression effect
- Data users should expect a higher level of confidence in the absolute scale of density/temperature measurements and notice less sinusoidal bias in the velocity X and Y components (GSE)

## 1 Introduction

The Magnetospheric Multiscale Mission (MMS) is a set of four spacecraft flying in a tetrahedron formation designed to study magnetic reconnection in the Earth environment, with applications to heliophysics, astrophysics, and fundamental physics (Burch et al., 2016). The MMS primary mission lasted 2 years, starting during the completion of commissioning in September 2015. The Fast Plasma Investigation (FPI) measures full‐sky ion and electron velocity distributions by counting particles with the Dual Electron Spectrometers (DES) and Dual Ion Spectrometers (DIS) (Pollock et al., 2016). In addition to FPI, the MMS instrument suite also provides measurements on the magnetic field, electric field, ion composition, and energetic particles of the surrounding environment (Mauk et al., 2016; Torbert et al., 2016 and Young et al., 2016).

The DES are the component of FPI which measure electron distribution functions. During the September 2015 to March 2016 duration (“Phase 1A”) of the primary mission, the mission recorded data between the inner side of the magnetopause and the outer side of the bow shock boundary. At this time, the DES suite was configured to generously utilize onboard lossy compression, meaning that much of the data was altered by compression error**.** In the modern age, the advancing instruments of scientific missions generate high‐resolution data at rates that outpace the capacity to transmit data to the ground. Payload data compression is a critical element of scientific spaceflight, as even the simplest compression algorithm will more than double the volume of data returned.

Lossy compression is a form of compression that drops information content in exchange for reduced data size. This contrasts with lossless compression, which reduces the file size while preserving the information exactly. Lossy compression for imagery often works by reducing spatial redundancy and dropping information the algorithm deems to be insignificant to the use case. Generally speaking, lossy compression outperforms lossless compression but pays with varying levels of consequence.

FPI also consists of the DIS. DIS frames are compressed with the same method as DES, although with a different tuning and a resulting different style of lost information content due to the nature of the data. The fundamental difference between the DES frames and DIS frames that preclude significant information loss for the DIS frames is twofold. First, DIS counts plasmas whose particle are clustered more tightly around their central direction, producing a type of distribution that works better for the compression. Second, DIS has a lower geometric factor which results in frames with less counts and reduced information entropy. In this paper, we will focus on the DES instrument correction only.

The compression is performed by a set of application‐specific integrated circuits (ASICs) collocated with the instruments. The ASICs are programmed to implement a compression algorithm known as the discrete wavelet transform (DWT) and bit‐plane encoder (BPE). This is a standard for space‐borne lossy image compression as published by the Consultative Committee for Space Data Systems (Yeh et al., 2005). The DWT/BPE algorithm has two modes, integer mode and floating‐point mode. It is parameterized by a maximum file size setting (bytes). When the bytes required to represent the images in its wavelet form exceeds the maximum frame size, the algorithm begins truncating data. In integer mode, it drops entire wavelets until the total file size is under the maximum frame size. In floating‐point mode, the trailing accuracy of wavelet coefficients are truncated until the file size is under the maximum file size.

The FPI suite's data budget is designed to downlink a small subset of its observation cycle at high‐time‐resolution and the remainder at low‐time‐resolution. The subset downlinked at high‐time‐resolution may come from any part of the observation cycle and is controlled by scientists on the ground, with the inclusion criteria guided by the mission's science objectives. The high‐time‐resolution data is known as burst mode data and the low‐time‐resolution data is known as fast survey data.

During the September 2015 to March 2016 period of Phase 1A, the DES compression was configured with integer mode and the maximum file size was set to a size that caused the method to truncate for 57% of the DES frames. This truncation leads to varying degrees of degradation in scientific quality. The science operations team controlling DES performed a trade‐study upon the completion of Phase 1A and reconfigured the ASIC for the next phase. This reconfiguration set a larger maximum file size for both fast survey and burst mode and set fast survey mode to use floating‐point mode. This reduced the truncation rate for burst mode to around 5%. Evaluation and further discussion of this reconfiguration are documented in Barrie et al. (2017).

The most significant issue associated with lossy compression on MMS/DES is the addition of nonphysical counts to specific regions of velocity distribution function (VDF) frames. These frames are created from the three‐dimensional (3‐D) VDF measurements by a reorganization of the frame (see section 2). Such additions have the largest impact on magnetospheric burst data, in which case the counts originating from the environment are on the same order as the nonphysical counts added by the compression algorithm. Moments of measured VDFs such as density, bulk velocity, and temperature can be distorted by these compression artifacts. Compression artifacts in DES tend to be focused in low‐count areas on the left side of distribution function frames (see Figure 17 in Barrie et al., 2017). As will be shown, these artifacts result in systematic underestimates and overestimates of temperature and number density, respectively. In addition, such artifacts result in unphysical spin‐modulations in the bulk velocity. These spin‐modulations are characteristically different than those induced by imperfect sensor suite flat‐fielding (Gershman et al., 2019) and create a discrepancy between fast survey and burst resolution moments (Barrie et al., 2017). As will be shown, elimination of compression artifacts in the DES data set can result in more accurate moments in the magnetosphere.

A detailed description of the correction method and evaluation follow in this paper. In section 2, a description of the compression‐related degradation is discussed, and examples are shown. In section 3, background is given on the data set of before‐and‐after frames we used to drive optimization of a correction model. In section 4, we describe the physics and structure of the model building using linear algebra. Section 5 relates the solution of the neural network optimization to basis functions and argues an interpretation based on mathematical principles.

Section 6 reinforces the interpretation with statistical evidence that the method is effective at reversing the compression effect. Finally, section 7 summarizes the findings presented in this paper and provides possible paths to improving upon this method.

Readers interested in reading a quick summary of the correction's improvement level may skip to section 6 and come back to read the methodology later. A visual summary of the improvement can be found in Figures 12, 13, 14, 15.

**Figure 1:**
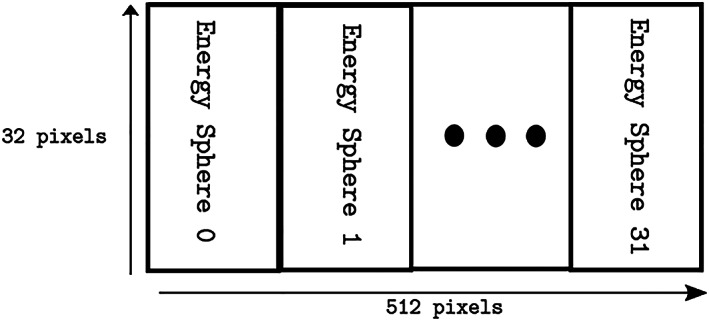
Layout of Distribution Function energy spheres into a single 2‐D image. Each energy shell, labeled above as ‘Energy 0’, ‘Energy 1’ and so on, has a size of 16x32 (width x height) with the width corresponding to the polar angle and the height to spin plane angle.

**Figure 2:**
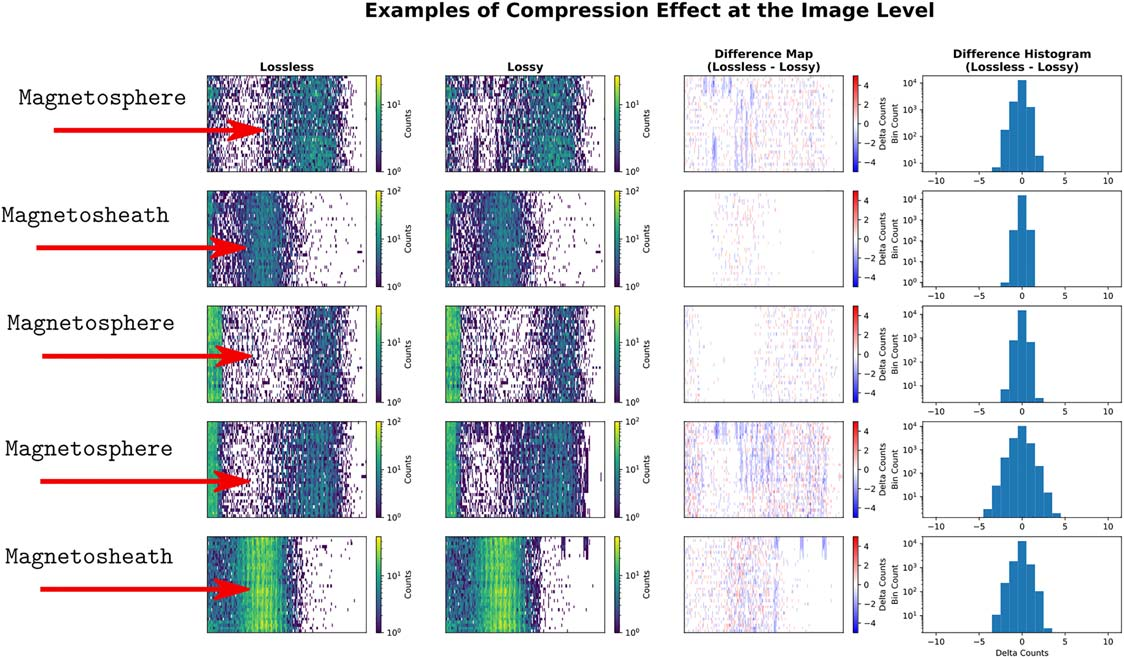
Examples of Compression Effect at the Frame Level. The pristine frames from the first column were compressed to generate the degraded images in the second column. The third column shows a difference map between the pristine and degraded frames, where‐in the ‘blotchy’ artifacts can be easily identifiable as the blue blobs. The difference histograms are shown on the right (fourth column), where‐in a skewed Gaussian pattern can be seen..

**Figure 3:**
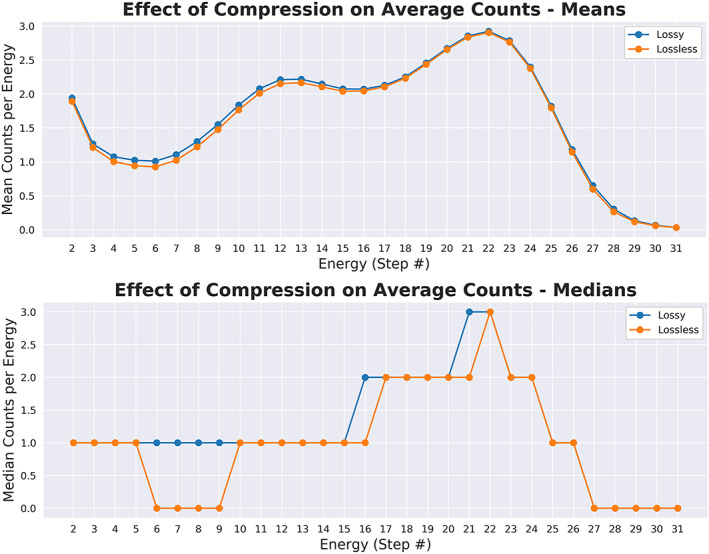
Compression and decompression caused counts to be added to the lower energies, found between the photo‐electrons (energy levels 0 and 1, not plotted) and typical magnetosheath thermal energies. By adding counts, the density was increased, and by adding them to this region, the temperature was decreased (Figure 4). Counts are low compared to Figure 2 because the photo‐electron contaminated energies are not included in this plot and the magnetosheath populations fail to dominate the averages.

**Figure 4:**
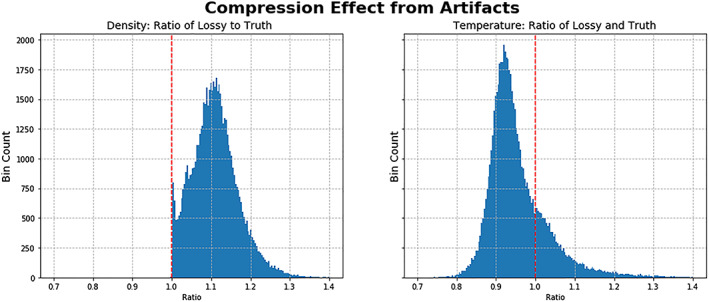
Compression and decompression caused a systematic increase in in density and decrease in temperature. Data from Phase 1B was processed into moments to create the ground truth, and processed as second time after compression and decompression to create the lossy version. The values being histogrammed here are the ratio of the two, with lossy on the numerator and truth on the denominator. A ratio of 1 corresponds to equal values and is ideal.

## 2 DES Compression Summary

The DWT/BPE compression algorithm standard requires a two‐dimensional (2‐D) image. DES counts particles over 3‐D discretization of velocity space, with one dimension spanning the particle's kinetic energy and the remaining two describing the travel direction in spherical coordinates. The three dimensions are reordered into two dimensions by separating the velocity space grid into spheres of equal energy, each of which is a 2‐D image. These 2‐D images are then placed side by side in left‐to‐right order from lowest to highest energy (Figure 1). Together, they form one 512 × 32 image (width × height). The bit depth of the pixels is 10 bits.

**Figure 5:**
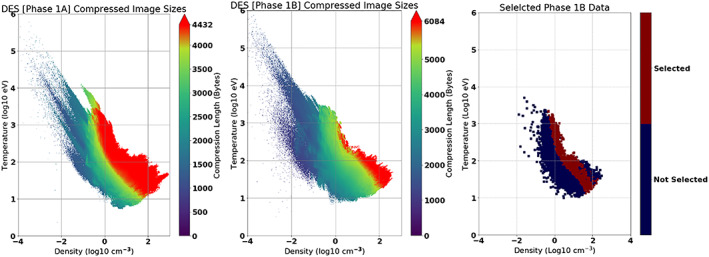
The compressed files sizes in Phase 1A and Phase 1B, and data selected from Phase 1B as training data In the left two plots, when the file size overflowed past the maximum allotted threshold, it was truncated and appears in the plot as red. Two conditions were required for the data to be chosen: it must have been predicted to have had lost information content if compressed under the Phase 1A rules, and it must also have been downlinked with no lost information content in Phase 1B. In the right‐most figure, the data on the top right was not selected from Phase 1B because it contained lost information content.

Typical histograms of velocity space from MMS mission in this format are shown in Figure 2. The conversion factor between the histogram value and the phase space density distribution function value is dependent on a combination of the geometric factor (Collinson et al., 2012) and detection efficiency for that bin's pointing direction. In addition to ambient particle counts, a population of instrument‐generated photoelectrons (Gershman et al., 2017) are typically found at the lowest two energy levels.

**Figure 6:**
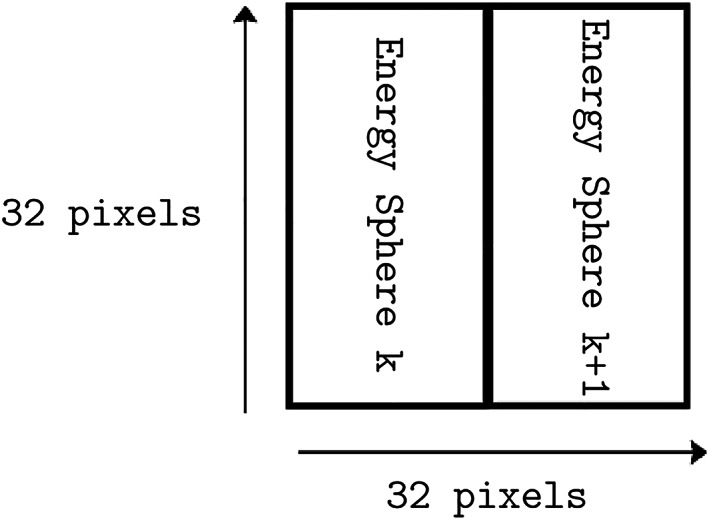
The 32x32 sub‐image on which the correction kernel acts. Each sub‐image contains two energy steps, allowing for inter‐energy information to be utilized.

A previous large‐scale empirical analysis of DWT/BPE on Phase 1A data from MMS displayed that the number of bytes the algorithm requires to encode a frame is a function of the number density and temperature (Barrie et al., 2019). It was observed that the higher the number density, the more bytes required, and the same for the temperature. This agrees with an information‐based model of the compression task: As the number density goes up, the difference between the minimum and the maximum pixel values increase accordingly, along with the number of possible values a pixel can take. As the temperature goes up, the horizontal spread of nonzero pixels across energies increases and the algorithm has less opportunity to encode large blocks of zeros with an abbreviation code. This is consistent with the general idea that compression efficiency scales inversely with image entropy.

A software implementation of the DWT/BPE written in C was used to emulate the hardware implementation used onboard MMS. Using the flexibility of ground‐based emulation, we compressed and decompressed several hundred thousand lossless frames to study the effect of the compression at both distribution level and the moment level.

At the frame level, the compression effect manifests in the form of both salt/pepper noise over nonzero regions and “blotchy” compression artifacts (see Figure 2). The salt/pepper noise showed an overall approximately Gaussian spread with various levels of skew and typically did not appear in regions of zero or very sparse counts. Though the spread was typically approximately Gaussian, the width of the distribution varied between frames.

“Blotchy” compression artifacts were introduced into regions of zero to low counts with pixel values of two to three counts. These can be seen in the difference maps below (third column) as the concentrated regions of blue. For magnetospheric data (low counts and lower signal‐to‐noise ratio), these artifacts were at approximately the same level as counts from the core population. For magnetosheath data (high counts and higher signal‐to‐noise ratio), these artifacts were not.

We sought to answer the question as to whether the compression effect increased or decreased the counts overall. To answer this, we plotted the average counts before and after compression at each individual energy level (Figure 3). We found that at the energy steps between photoelectron/potential noise (energies 0 and 1) and the typical magnetosheath thermal speed (around energy 10) counts were systematically added, but this effect was not present at the other energy steps.

**Figure 7:**
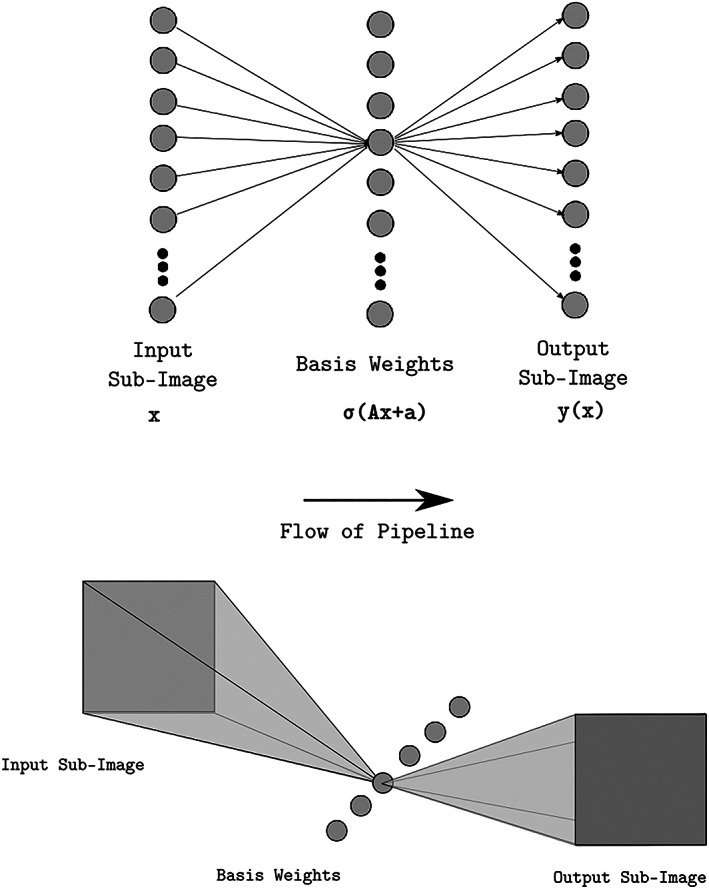
Top: the neural network architecture diagrams, in two forms. The top diagram is the typical way to display a multi layer perceptron neural network. Each column is a vector, referred to as a layer. Though only the lines connecting to one basis weight are drawn, they exist for all basis weights. The diagram displaying the input and the output as images with an analogy with geometric optics.

It was not clear why the higher energies were less effected. One possibility is that when forced to truncate the wavelet expression, the compression algorithm picks wavelets to drop first from those at the lower energies. If this were true, it is possible that often by the time the maximum file size is achieved, the higher energies were left untouched.

At the moment level, we launched a statistical analysis difference in moments between the ground truth points and the compressed/decompressed counterparts. We observed that the compression effect propagated into higher densities and lower temperatures (Figure 4). The higher densities are consistent with the overall increase in counts as the increased led to believed particle detections which did not occur. The lower temperatures are consistent with the location at which the counts are added, in addition to counts with speeds lower than the true thermal speed pulled the average down.

**Figure 8:**
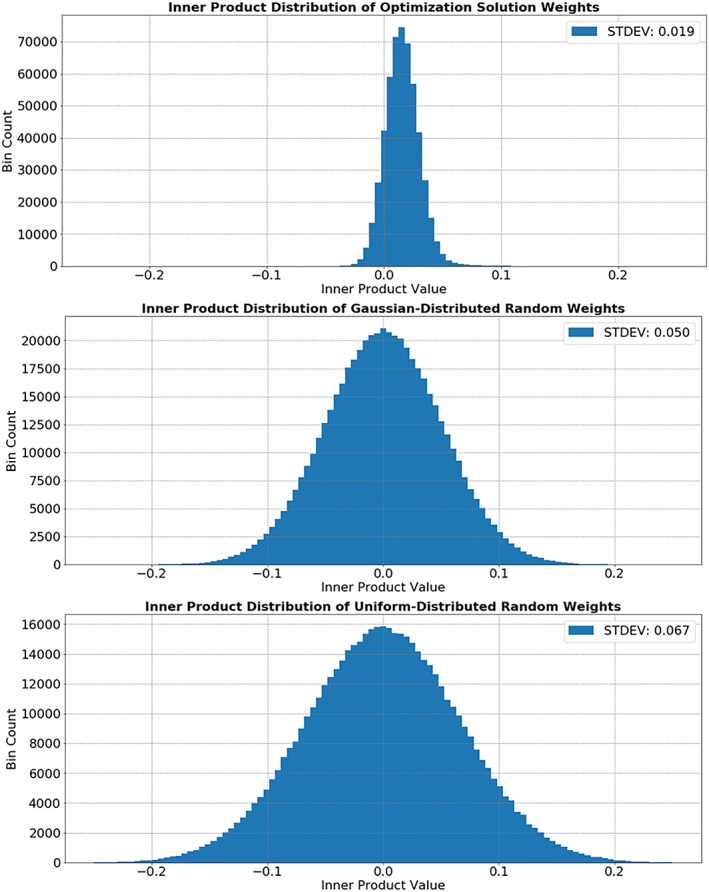
Inner production distributions for basis functions from optimization solution, random sampled basis functions from Gaussian distribution, and random sampled from uniform distribution (each over same scale as solution). The result that the basis functions for our optimization solution are more orthogonal supports our description of the columns of *B* as basis functions. The center inner product value being slight positive is expected to arise as a result of the b bias vector in the equation.

Regarding the bulk velocity, we noticed an additional sinusoidal variation in the spin‐plane components (approximately X and Y within a few degrees) in the data on top of the existing sinusoidal variation (spintone) caused by natural inaccuracies in the flat‐fielding calibration (Gershman et al., 2019). These two sinusoidal variations constructively interfere and hide time‐variation in the underlying data. The variation in the bulk velocity is understood as persistent blotchy artifacts in one area of the frame that add bias weight to the bulk velocity integral in a direction stationary within the spacecraft frame. The spin‐axis component (approximately Z within a few degrees) was largely unaffected, understood to be due to the cycling of the polar dimension across the image width.

## 3 Training Data Used to for Model

Following this period of lossy compression, the DES was reconfigured the next phase, Phase 1B, in a way that allowed for 95% of the frames to arrive at the ground without loss. Using this set of frames from on‐orbit observations, we compressed and decompressed it on the ground to create a side‐by‐side record of the compression effect to use for training a model.

The criteria for measurements from Phase 1B to be included in the training set are modeled as needing to meet two conditions. The first condition (1) is that it must be highly likely to produce lost information content when subjected to the compression algorithm rules from Phase 1A. The second condition (2) is that in its existing form it must have zero lost information content through the compression algorithm acting in Phase 1B. These are both required so that the measurement may be useful for yielding a strong side‐by‐side example of the effect.

The property of lost information content in Phase 1A was found to appear strictly within a connected subset of density‐temperature space. An algorithm to implement condition (1) was achieved drawing a boundary in density‐temperature space between the data predicted to have lost information content in Phase 1A and the data predicted to not. When we choose data on one side of the boundary from Phase 1B, we can produce a set that meets our first condition. To meet condition (2), we manually subtract out points whose file size is below the Phase 1B maximum file size.

The creation of the boundary was achieved using a polynomial support vector machine model (Trevor et al., 2009), a statistical method that solves for an optimal parametric border to separate differently labeled populations. Other more complex versions of support vector machines, such as those that support nonparametric boundaries, were not necessary. Another use of the parametric support vector machines to classify subsets of density‐temperature space for plasma species can be found in da Silva et al. (2020). The specific variation which we used was implemented in the open‐source package Scikit‐learn for Python (Pedregosa et al., 2011).

The class of desirable data from density‐temperature space is visually the red region from Phase 1A minus the red region from Phase 1B in Figure 5, highlighted in the rightmost plot.

**Figure 9:**
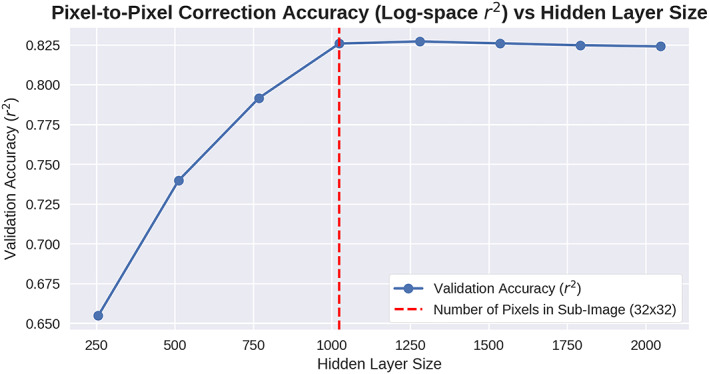
Results of experiment comparing validation accuracy metric versus hidden layer size. The validation metric is the r squared value between the true and corrected pixels. The hidden layer size is the number of basis functions, also known as the columns of C. The accuracy stopped increasing after the number of basis functions reached the dimensionality of the output space (the sub‐image). It is believed that limitations in the inherent predictability of the task, combined with limitations of the training method, prevent further accuracy after this point.

Using this criterion for whether a point was desirable, we selected burst intervals for which over 90% of the points in the interval met the criteria and the average density was under 5 cm^−3^. The density condition was added during a later iteration of model development, after recognizing that frames corresponding to very dense plasmas were dominating the model optimization, and they needn't be explicitly represented in order to successfully learn to correct them.

Using the ground‐based emulation of the onboard DWT/BPE, later phase data that would have been lossy in the Phase 1A configuration were compressed and decompressed to create the side‐by‐side examples.

## 4 The Neural Network Architecture

The neural network is a general‐purpose nonlinear function approximation tool which learns the appropriate representation of a function for examples of its input and outputs. Neural networks have previously been applied in the area of space physics instrumentation by way of the FPI pseudo moments correction (Barrie et al., 2018). Machine learning in the context of space physics/weather has been discussed extensively in the literature in recent years (Camporeale, 2018; Camporeale et al., 2019; McGranaghan et al., 2017; Yang et al., 2018).

The side‐by‐side records of the compression effect provided information to train a neural network to perform the complex, nonlinear correction task. Without a large set of side‐by‐side records to train general‐purpose model, a hand‐crafted approach would have been required, which is traditionally much less effective.

The correction task was designed to act on one frame at a time, with the correction kernel mapping localized compressed 32 × 32 subimages of the frame as an image to their corrected 32 × 32 subimage counterparts in log‐space (Figure 6). A 32 × 32 subimage consists of two consecutive energy spheres. The design of this subimage was based on the observation that the information required to identify and remove an artifact is localized to the neighborhood of the artifact itself, with the definition of neighborhood agreeing with viewpoints of the frame as 3‐D count map and also a 2‐D pixel image. The use of log‐space for the prediction was chosen as to not overpenalize the optimization process in denser plasma regimes.

**Figure 10:**
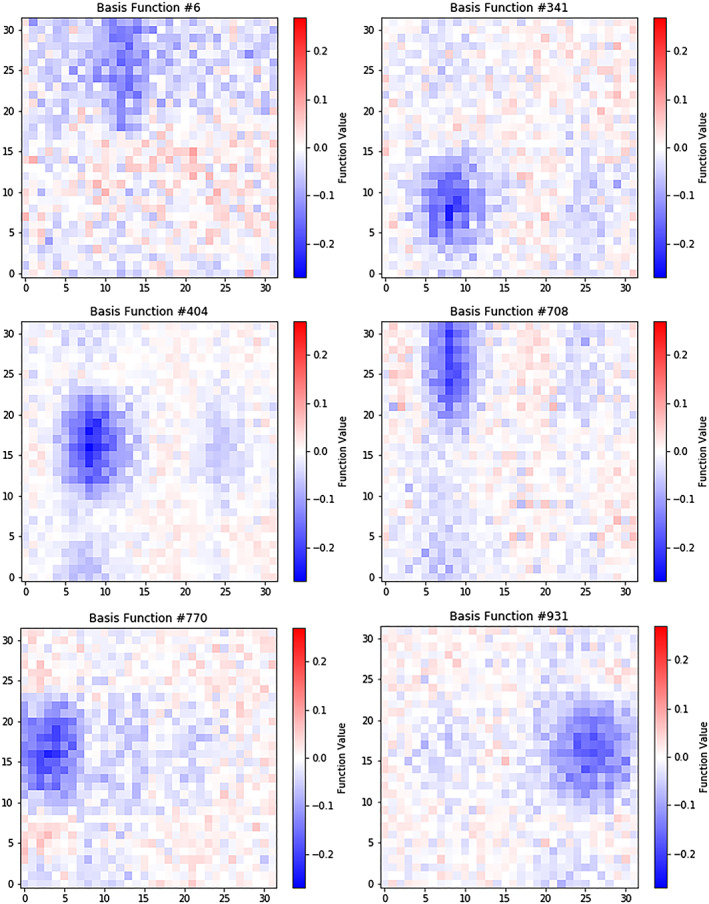
A set of pattern‐based basis functions. These functions place or subtract ellipse‐like patterns on the image, in a behavior that is reminiscent of spherical harmonics. The images show here represent about 50% of the total pattern‐based basis functions of this class.

The general‐purpose model chosen to meet the requirements of the correction kernel was the multilayer perceptron, which, when trained, arrives at a set of 2‐D basis functions capable of approximating the sample space, and a corresponding function that maps the lossy image to nonnegative basis weights that recreate the image without compression artifacts. This image is then converted to a frame by unraveling it back into a 3‐D structure.

This can be implemented as a neural network, under which the method has been previously studied and proven for similar image denoising tasks under the banner of image processing (Burger et al., 2012). Further linkages into the realm of neural network research can place this as a denoising autoencoder, for which the first stage encodes the image into basis weights (the hidden layer) and the second stage decodes those basis weights into a final image.

The form of the general‐purpose equation that represents the correction kernel that maps 32 × 32 subimages to 32 × 32 subimages is (Table 1):

$$y\left(x\right)=C\sigma \left(\mathrm{Ax}+a\right)+c.$$

**Table 1:** Variables and Functions Included in the Multilayer Perceptron Neural Network Model Equation

Variable	Type	Size	Description
*y*(*x*)	Vector	1,024 (=32^2^)	The corrected subimage flattened into a 1‐D vector, in log‐space
*x*	Vector	1,024	The compressed subimage flattened into a 1‐D vector
*C*	Matrix	1,024 × 1,024	The columns of this matrix correspond to the basis functions used to represent the corrected image
*c*	Vector	1,024	The offset applied after the corrected image is approximated with basic functions
*A*	Matrix	1,024 × 1,024	The rows of this matrix represent the linear combination of input pixels used to calculate the basis weights
*a*	Vector	1,024	The offset used in the calculation of the basis weights
*σ*	Vector‐to‐vector Function	1,024 ➔ 1,024	The function **σ(α) = max(α, 0)** applied over each element of its input vector. Prevents basis weights from being negative by replacing negative values with zero.

*Note*. The variables A, a, B, and b are optimized during the training phase. The **σ** function replaces negative arguments with zero.

This equation is often represented visually in the classic neural network diagram with the first column the input vector **x**, the middle column the basis weights ***h***(***x***) ***= σ***(***Ax + a***) **,** and the final column the output vector **y(x)** (Figure 7)**.** The middle column, ***h***(***x***), is known as the hidden layer as its values are typically not used as output. The matrix multiplication in this diagram is expressed with a series of arrows with the source of the arrow connecting to the value the weight multiplies, and the destination is the value to which the product contributes to a sum.

**Figure 11:**
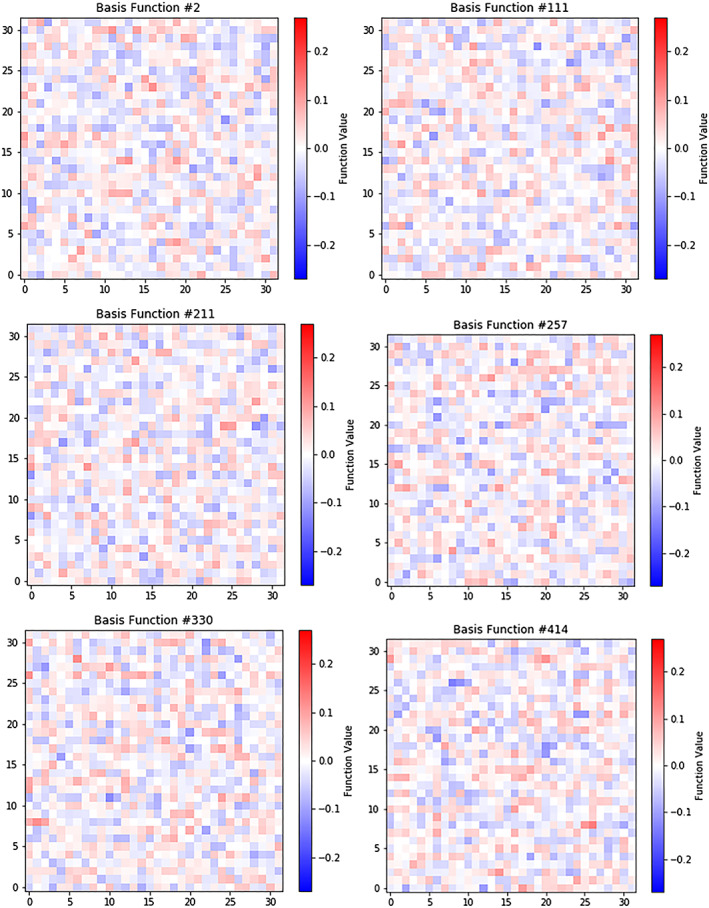
Salt/Pepper Noise Basis Functions. These basic functions appear as static buzz, with no discernable structure. However, they are statistically orthogonal to each other, and support the ability to reconstruct the image including noise.

The columns of the matrix **C** multiply the basis weights and correspond to 2‐D discrete basis functions that represent the image. When these columns are reshaped into 2‐D images, they become constituents that are added together, along with the final bias **c**, used to reconstruct the corrected image. An analysis of these basis functions shows that their inner product distribution is more clustered around zero than if their values were sampled randomly at the same scale. This is demonstrated in more detail in section 5.

The term of the equation stated as **σ (Ax + a)** determines the values of the basis weights based on the input image. This can be interpreted as each basis weight being a linear combination of the input pixels plus a bias, with the after‐computation effect of being set to zero when it is negative. The σ function, called the activation function in the body of neural network research, replaces the negative value with zero and served the purpose of introducing the nonlinearity capability into the system. Without this σ function, the neural network would reduce to a linear system of form **C (Ax + a) + c** to **Dx + d** and be significantly more limited. Further discussion the activation function and how it enables nonlinear structure can be found in Trevor et al. (2009).

Postprocessing on the neural network output involves scaling the prediction downwards to account for overestimation in the neural network output. Because the predictions are done in log‐space, overestimates are penalized less than underestimates, which incentivizes neural network training to converge on a function that overestimates. We found that scaling down by a factor of ½ was an effective correction factor to account for the tendency to overestimate values in the prediction. In addition to this, we restricted overwriting the lossy image only in the range of counts we know the compression artifacts appear (regions of two to three counts), though in log‐space, the compression errors are most dramatic in low density, high temperature plasmas.

## 5 Interpretation of Training Solution

Interpretation of the training solution lead to the observation that the training created notably orthogonal columns of **C.** These columns are henceforth referred to as the solution basis function. We argue them to be “notably orthogonal” by comparing their orthogonality to the orthogonality of random basis functions.

We calculated the distribution of inner products between the solution basis functions and a set of random basis functions sampled uniformly between ±2 standard deviations of the solution distribution which itself is Gaussian. To be certain, we also compared with random basis functions sampling from a Gaussian distribution with the same location and shape as the solution distribution.

The inner product used was the standard inner product as defined for vectors:

$$<\vec{x},\vec{y}>=\sum_{i=1}^{N}x_{i}y_{i}.$$

This can be computed quickly through the off‐diagonal elements of *C*^*T*^*C* (the diagonals correspond to the inner product of a basis function with itself). This is a discrete analog to the standard continuous function inner product defined as

$$<f,g>=\int_{a}^{b}f\left(t\right)g\left(t\right)dt.$$

The distribution of inner product values for the optimization solution and the random samplings can be seen in Figure 8. The result that the basis functions for our optimization solution are more orthogonal supports our description of the columns of **C** as basis functions. The inner product distribution for the optimization solution had a standard deviation of 0.019, while the uniform random sampling had a standard deviation of 0.050, and the Gaussian distribution had a standard deviation of 0.067.

**Figure 12:**
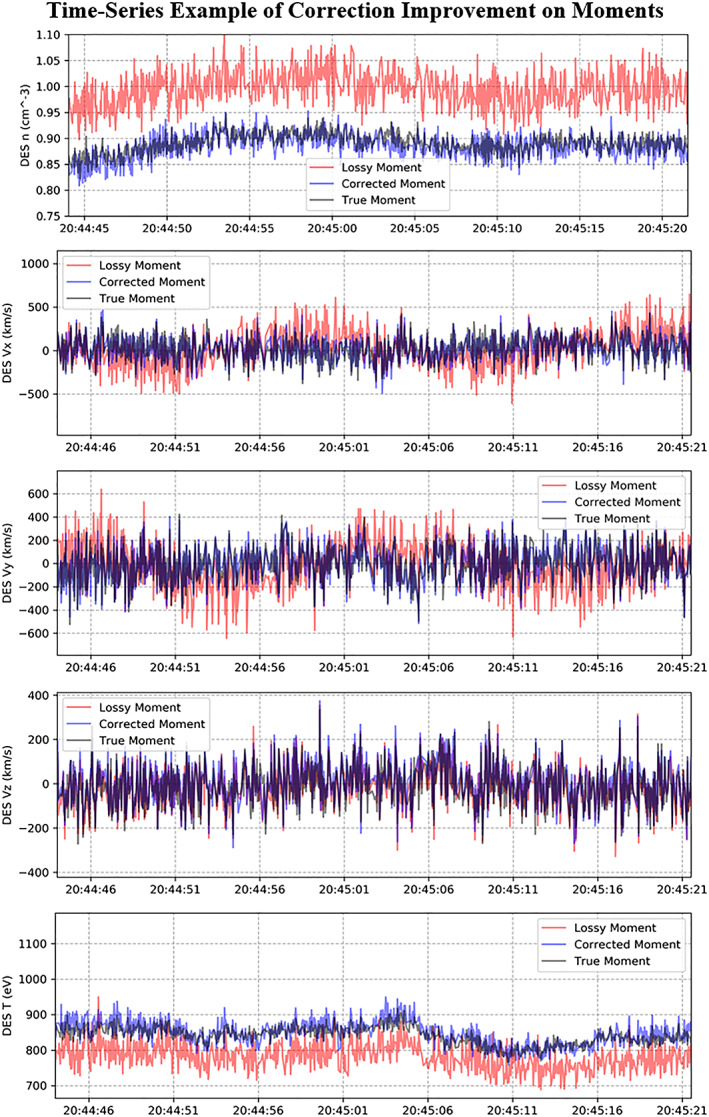
Example of time series evaluation of the correction. The density and temperature values were shifted with the shape staying the same. In the velocity components, primarily the X and Y velocity components (GSE), a sinusoidal bias was removed. This data is taken from the MMS4 interval starting at 2016‐10‐10 at 20:44:44 UTC.

Further evidence for the description of the columns of **C** as basis function comes from an experiment preformed to reveal the best performing number of columns of **C** in the neural network equation. The number of columns in **C**, referred to in the neural network literature as the hidden layer size, controls the number of basis functions created and utilized.

In our experiment, we trained models with a hidden layer size at the levels between 25% to 200% (inclusive) of the nominal subimage size of 32 × 32 = 1,024. We compared these models by testing their performance on correcting subimages outside of the training set using the accuracy metric of correlation coefficient between the true and corrected pixels. An analysis of the accuracy metric versus the hidden layer size (Figure 9) showed that the accuracy stopped increasing after the number of basis functions was approximately the number of pixels in the subimage (1,024).

**Figure 13:**
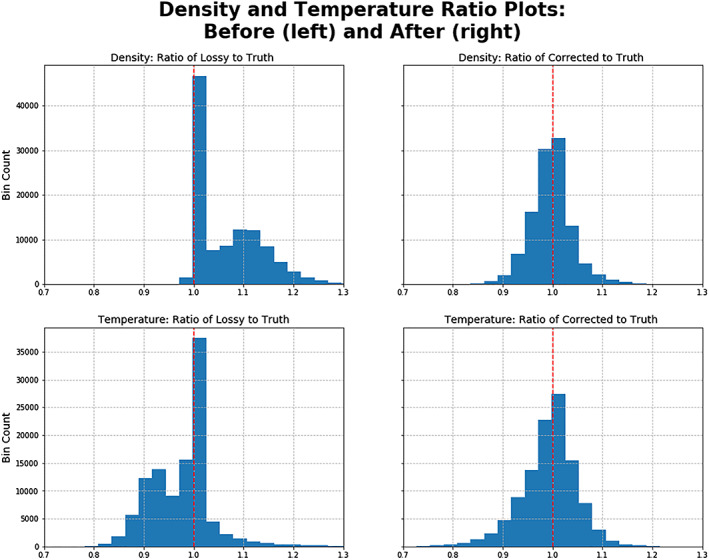
Histograms of lossy moment over true moment and corrected moment over true moment, for moments of density and temperature. After the correction process, the histogram centered its expected value around 1, indicating that on average the corrected moment was equal to the true moment. The population around 1 for the ratio of the lossy density moment to true density moment was from images where the compression effect created salt and pepper noise but not artifacts.

This is consistent with the linear algebra interpretation of reconstructing a vector using basis vectors. In linear algebra, any vector in an N‐dimensional space can be reconstructed using a linear combination of N basis vectors provided these basis vectors are linearly independent. That is, a set of N linearly independent basis vectors in an N‐dimensional space is sufficient to span the entire N‐dimensional space.

The basis functions were visualized to gain insight into how the image is reconstructed. To do this, we reshaped the columns of **C** into 32 × 32 images and plotted them. In this form, each of the 32 × 32 basis function images are scaled by their basis weight and added together, along with the final bias **b**. In addition to the basis functions, we reshaped the vector bias **b** into a 32 × 32 image and plotted it.

The plots showed that there are two classes of basis functions—ones that consist of pattern‐based weightings (approximately 5% of all basis functions) and those that consist of primarily of salt/pepper noise (approximately 95%). Pattern‐based basis functions showed values in patterns which can be interpreted as having interpretable physical significance.

The pattern‐based basis functions show behavior reminiscent of spherical harmonics (Figure 10). They placed or subtracted shapes on the image at different location, with the most common shape being an ellipse at a particular location.

**Figure 14:**
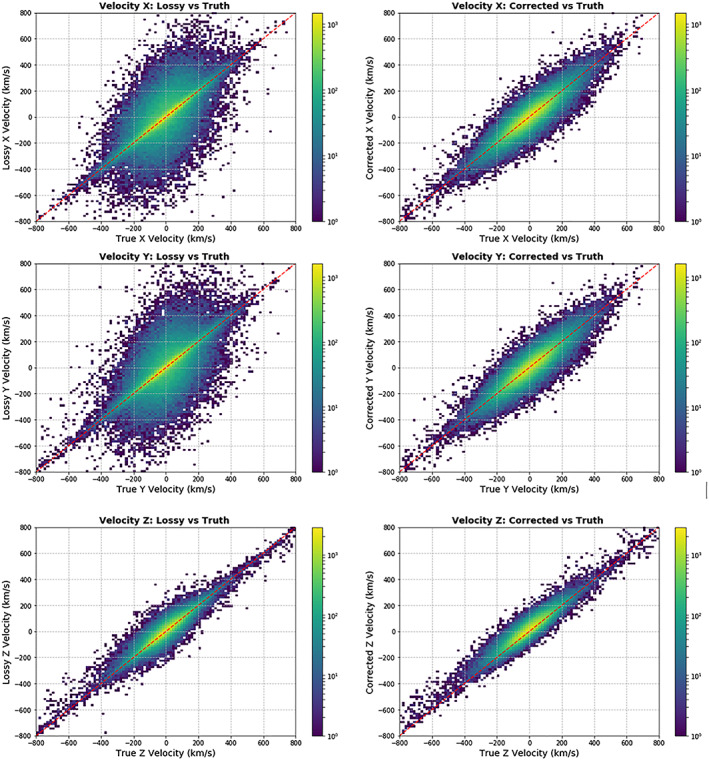
Two dimensional histograms of the velocity before and after the correction (N=110,00). On the left column, the y‐axis shows the lossy version. On the right column, the y‐axis shows the corrected moment and the true moment. In both columns, the x‐axis shows the true moent. The spread of the points over the y‐axis before the correction (left column) is much larger than the after the correction (right column) for the X and Y moments, indicating the correction is working.

The salt/pepper noise basis functions appeared as static buzz with no discernable structure (Figure 11). Though these basis functions do not have a discernable structure, they proved orthogonal to each other overall, which indicates that they support reconstruction capability.

**Figure 15:**
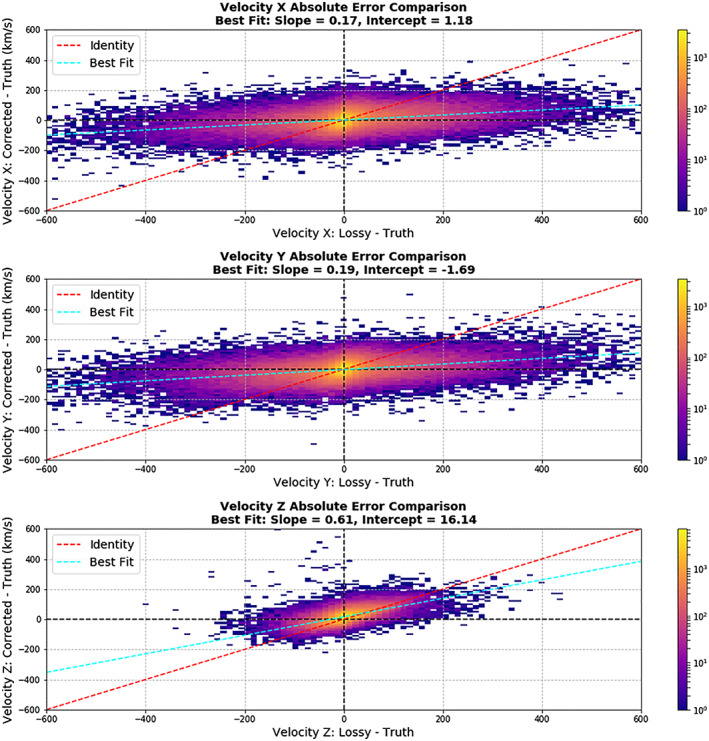
Error by error comparison between the lossy residual (between lossy and true moment) and the corrected residual (between corrected and truth). The slopes of .17, .19, and .61 (X, Y, and Z velocity respectively) represent the residual was on average reduced to 17%, 19%, and 61% of its original value, a significant improvement

The authors believe that the imbalance in pattern‐based basis functions (5%) and salt/pepper noise basis functions (95%) has to do with the difference in their roles when the image is reconstructed. In our understanding, the pattern‐based basis functions reconstruct the high‐level structure, while the salt/pepper basis functions reconstruct the low‐level noise. Previous studies have shown that relatively few spherical harmonics basis functions are required to reconstruct moments from phase space density measurements (Viñas & Gurgiolo, 2009). If the pattern‐based basis functions are playing a role like spherical harmonics, this would imply that the complexity of representing the noise is much greater. This fact would be consistent with traditional signal processing theory (Shannon, 1948).

## 6 Evaluation

Evaluation of the method showed a shift from asymmetric/atypical error distributions to Gaussian error distributions (for the density and temperature moments). The corrected velocity X and Y components (GSE) showed a large improvement by removing a sinusoidal bias over a spin phase (20 s). There were smaller improvements in the velocity Z moment (GSE).

A discussion of the evaluation follows. This method was evaluated statistically and visually on an independent set of side‐by‐side records not used for model optimization. This evaluation was performed both qualitatively and quantitatively.

In section 6.1 are moments plots of the before, after, and true moments presented in the way one would traditionally review FPI data. In section 6.2, histograms of the before and after moments to the true moments are compared. In section 6.3, colored‐by‐concentration 2‐D histograms compare the before and after moments to the true moments. In section 6.4, the before and after errors are plotted and summarized quantitatively.

### 6.1 Evaluation at the Time Series Level

In the time‐series plot evaluation, one can see improvement in the quality of the moments (Figure 12). The scale of the density and temperature is adjusted back to the true value, while their general shape over time is retained. In the velocity components, primarily the X and Y GSE velocity components, a sinusoidal bias is removed. This sinusoidal bias was caused by an artifact appearing in the same location in the spacecraft image, which rotates along with the spacecraft.

### 6.2 Evaluation at the Density and Temperature Ratio Level

The overall temperature and density are improved on average, as seen in Figure 13, which includes points that were lossy but did not produce severe artifacts. The distribution of ratios before was asymmetrical and indicative of non‐Gaussian uncertainty. After the correct, the ratios became more Gaussian.

### 6.3 Evaluation at the Velocity Point‐by‐Point Level

The velocity components showed a large improvement after the correction effect when the components are plotted on a point‐by‐point basis (Figure 14). In this evaluation, a scatterplot‐like evaluation of 110,000 points we see that when we compare the lossy moment to the true moment, we find it has much higher uncertainty than when we compare the corrected moment to the true moment. This indicates a large‐scale improvement.

### 6.4 Evaluation at the Velocity Error Point‐by‐Point Level

A second evaluation shows a point‐by‐point fit between the lossy residual (between lossy and true moments) and corrected residual (between corrected and true moments). In this analysis, a linear fit was done between the two errors. We found that for the X and Y velocity moments (GSE), the residual was on average reduced to 17%, 19%, and 61% of its original value (X, Y, and Z, respectively). This is labeled as the slope of the fit. The intercept of the fit represents the approximate average increase or decrease in residual when the lossy residual is zero. This was 1.18, −1.69, and 16 km/s (X, Y, and Z, respectively), which is close to zero. It is noted that the Z bias is larger than the others, and this is accepted in exchange for the large residual reduction.

### 6.5 Evaluation at the Frame Level

**Figure 16:**
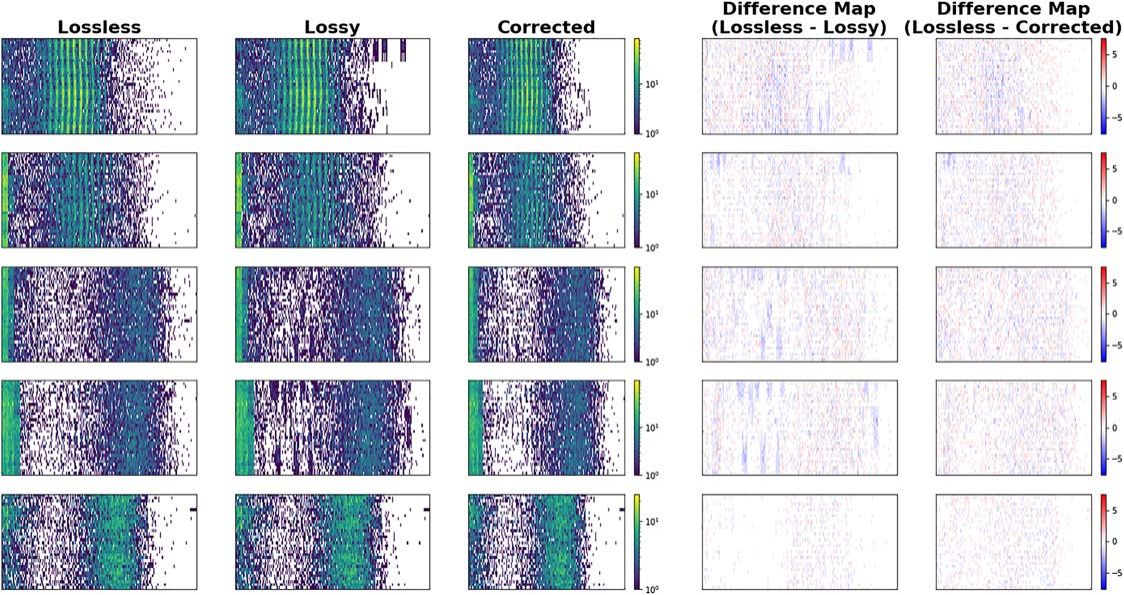
Example of correction process at the image level. Each row corresponds to a single frame in three states ‐‐ ideal uncompressed (lossless), after the compression effect (lossy), and after the correction (corrected). The artifacts are the blue blotches appearing in the second‐to‐right column (difference map between lossless and lossy). After the correction (right most column), these blotches disappeared.

The evaluation at the image level visually confirms that the artifacts were removed (Figure 16). In this evaluation, difference maps were plotted before and after the correction and manually identified the presence of artifacts which appear as blue blotches in the difference maps below. After the correction, the artifacts disappeared. There is a slightly higher level of salt/pepper noise in the corrected image than the lossy image, but this type of noise does not contribute to uncertainty in the moments systematically.

## 7 Conclusion and Recommendations

The Phase 1A FPI data set was repaired from a period of damaged quality caused by lossy compression. With this correction, burst‐resolution data from the period of September 2015 to March 2016 of the FPI data set can be interpreted with higher confidence. Data users should expect a higher level of confidence in the absolute scale of density/temperature measurements and notice less sinusoidal bias in the velocity X and Y components (GSE). This repaired data set is not yet broadly applied or available, but the correction is now possible.

In this paper, we integrate techniques from fundamental image processing and computer vision research into experimental space plasma physics. A 3‐D distribution of velocity space counts can be rolled into a 2‐D image under various schemes, one of which was presented in this paper. When reshaping the form of the structure from 3‐D to 2‐D, regardless of scheme, one must carefully consider the way one redefines the implied meaning of neighboring cells. As seen with the DWT/BPE compression algorithm, artifacts bled between neighboring cells in the 2‐D form, a relationship that does not transfer logically to the 3‐D form.

We utilized a fundamental tool of modern image processing—the neural network—to solve a very complex and nonlinear problem for which a rule‐based solution would be extremely difficult, if not impossible. Interpretation of the underlying mathematics of the neural network provided an elegant solution based on basis functions that gives way to a meaning physical interpretation of the reconstruction process. With this methodology, the image repair and reconstruction process can be analyzed with a conventional mathematical toolset.

Through a multistep validation approach, we demonstrated the corrected data improves the measurements as desired. First, the frames were visually confirmed to remove the artifacts as expected. The corrected frames were processed through the MMS/FPI moments processing software, and it was found that the compression bias to the moments was largely reversed in the effected density, temperature, and velocity X and Y moments. Comparisons were made through plots of time‐series and through aggregated statistics. Correlating the before and after velocity X and Y residuals confirmed that the residuals were drastically reduced. The relative error in the density and temperature moments before and after the correction was analyzed, displaying that the correction turns the non‐Gaussian uncertainty distribution into a Gaussian one. Through this multistep validation approach, we deeply vetted the correction method and found it to be successful for producing science‐quality data.

While we have corrected the data for lossy compression artifacts, the ideal solution for mission system designers remains to preventatively mitigate systematic biases from lossy compression before launch as much as possible. For missions that experience this problem and are unable to adjust existing flight hardware, a solution to recover the data may be available.

Much quality research has been done by the computer image processing community on reversal of image artifacts, denoising, and image restoration. Several paths in improving this method could be explored.

During training, the orthogonality of the basis functions arose naturally. However, variations in the neural network strategy used here have been researched which impose strict basis function orthogonality by adding an additional penalty term to the error function. This penalty term takes the form of as below (where **B** is as is in our equation, **α** is a scale factor to tunes the penalty strength, and **tr(X)** is the trace of a matrix). Further development of this method could take the form of experimentation to enhance the correction's capability and interpretation.

$$\text{OrthogonalityPenalty}\left(B\right)=\alpha \left[\sum_{i,j}{\left(B^{T}B\right)}_{\mathrm{ij}}-\mathrm{tr}\left(B^{T}B\right)\right].$$

In addition to this, methods utilizing denoising with convolutional neural networks could be explored. Though their capabilities for interpretability differ from the technique utilized here, they are a powerful method and remain at the forefront of denoising research.

This study introduces a technique to process the 3‐D counts product from plasma instruments to remove a difficult to manage source of noise. Through successful application of a new technique, the door has been opened for transfer of similar image processing techniques to 3‐D counts products. Through additional capability building such as done here, additional complex and nonlinear noise issues can be addressed with on‐the‐ground data correction.
